# Integrative and complementary practices in Primary Care: unveiling health promotion*

**DOI:** 10.1590/1518-8345.3162.3277

**Published:** 2020-06-08

**Authors:** Indiara Sartori Dalmolin, Ivonete Teresinha Schülter Buss Heidemann

**Affiliations:** 1 Universidade Federal de Santa Catarina, Florianópolis, SC, Brazil.; 2 Scholarship holder at the Conselho Nacional de Desenvolvimento Científico e Tecnológico (CNPq), Brazil.

**Keywords:** Complementary Therapies, Health Promotion, Health, Family Health, Primary Health Care, Health Personnel, Terapias Complementares, Promoção da Saúde, Saúde, Saúde da Família, Atenção Primária à Saúde, Pessoal de Saúde, Terapias Complementarias, Promoción de laSalud, Salud, Salud Familiar, Atención Primaria de Salud, Personal de Salud

## Abstract

**Objective:**

to understand the use of integrative and complementary practices as a health promotion action.

**Method:**

qualitative study, action-participant type, with the application of Paulo Freire’s Research Itinerary, in which 30 Primary Health Care professionals participated. Thematic research was developed with two Primary Care Units, one that used integrative and complementary practices in daily life and another that focused more on allopathic concepts of assistance. To carry out the three stages of the method used, seven Culture Yarning Circles took place. The critical unveiling took place concurrently with the participation of those surveyed.

**Results:**

integrative and complementary practices constitute a form of health care, with the purpose of understanding the human being in the health-disease process, making it possible to work with the different aspects that involve them. In this way, they reduce damages resulting from the excessive use of medications, stimulate comprehensiveness and promote health.

**Conclusion:**

integrative and complementary practices are resources for health promotion, through comprehensive care and reducing the use of medications.

## Abstract (pt)

**Objetivo:**

compreender a utilização das práticas integrativas e complementares como ação de promoção da saúde.

**Método:**

estudo de abordagem qualitativa, do tipo ação-participante, com a aplicação do Itinerário de Pesquisa de Paulo Freire, no qual participaram 30 profissionais da Atenção Primária à Saúde. A investigação temática foi desenvolvida com duas Unidades de Atenção Primária, uma que utilizava as práticas integrativas e complementares no cotidiano e outra mais centrada nas concepções alopáticas de assistência. Para a realização das três etapas do método utilizado, ocorreram sete Círculos de Cultura. O desvelamento crítico aconteceu concomitantemente com a participação dos pesquisados.

**Resultados:**

as práticas integrativas e complementares constituem uma forma de cuidado à saúde, com a finalidade de compreender o ser humano no processo saúde-doença, possibilitando trabalhar com os diferentes aspectos que o envolvem. Dessa forma, reduzem danos decorrentes do uso excessivo de medicamentos, estimulam a integralidade e promovem a saúde.

**Conclusão:**

as práticas integrativas e complementares são recursos para a promoção da saúde, por meio da integralidade do cuidado e da redução do uso de medicamentos.

## Abstract (es)

**Objetivo:**

comprender el uso de prácticas integradoras y complementarias como una acción de promoción de la salud.

**Método:**

estudio cualitativo, tipo acción-participante, con la aplicación del Itinerario de Investigación de Paulo Freire, en el que participaron 30 profesionales de Atención Primaria de Salud. La investigación temática se realizó con dos Unidades de Atención Primaria, una que utilizó prácticas integradoras y complementarias en la vida diaria y otra que se centró más en las concepciones alópatas de asistencia. Para llevar a cabo las tres etapas del método utilizado, se organizaron siete círculos culturales. La presentación crítica se dio de manera simultánea con la participación de los encuestados.

**Resultados:**

las prácticas integradoras y complementarias constituyen una forma de atención de la salud, con el propósito de comprender al ser humano en el proceso salud-enfermedad, permitiendo trabajar con los diferentes aspectos que lo involucran. De esta manera, reducen los daños resultantes del uso excesivo de medicamentos, estimulan la integralidad y promueven la salud.

**Conclusión:**

las prácticas integradoras y complementarias son recursos para la promoción de la salud, a través de la atención integral y la reducción del uso de medicamentos.

## Introduction

The biomedical model is found in the current of positivist thought, supported by the technological apparatus, specialized knowledge and, consequently, the fragmentation of the human being^(^ 1 ^)^. The contributions of this model in reducing suffering caused by pathological events are undeniable, with effective action in a short period of time. However, health system professionals and users worldwide are realizing and becoming aware that the biomedical model does not have all the answers^(^ 2 ^)^, proving to be insufficient to meet the needs of population in different life cycles, opening space for a new health care paradigm. In this logic, integrative and complementary practices (*Práticas Integrativas e Complementares*, PIC) emerge from a perspective of placing the individual at the center of the process and all factors involved with it are scored at the time of therapeutic choice, prioritizing quality of life^(^ 3 ^)^.

The field of PIC includes complex systems and ancient therapeutic resources, transmitted from generation to generation. Researchers identified a gap between familiar/popular and scientific knowledge, revealed by the users’ fear of exposing the forms of care used, when they encounter health professionals in different institutions, which makes it difficult to monitor health^(^ 4 ^)^.

These, because they are in constant interaction with the population and users of the Brazilian Public Health System (Sistema Único de Saúde, SUS), have the role of offering alternatives to complement allopathic treatment, promoting health, preventing diseases, providing holistic care, with respect to beliefs, values and individualities^(^ 5 ^)^.

The National PIC Policy in Brazil seeks to develop training and qualification strategies in these practices for health professionals working in SUS, especially in Primary Health Care (PHC), in order to expand the forms of care and cure^(^ 6 ^)^. Therefore, it is essential to stimulate changes in health services, based on reflections on the work process, polishing concepts and habits, in order to modify the look and culture immersed in health, adding to professional understanding, popular and familiar knowledge and practices. Health promotion needs to be understood as a guiding axis, opening space for PIC, which emerge as a form of care that seeks empowerment, autonomy, comprehensive care and, promotion of individual, family and social health^(^ 7 ^)^.

Health promotion, in the PIC approach, requires rethinking the meaning of human beings’ autonomy in their ways of living, consolidating themselves in daily life, in schools, churches, businesses, leisure areas, health services, non-governmental organizations. Bearing in mind that the population, in general, uses different spaces and strategies in search of what traditional medicine does not provide: relaxation, support, moments of well-being and meeting with his self^(^ 8 ^)^.

An integrative literature review, which identified and analyzed the productions about PIC in PHC and its relationship with health promotion, revealed the lack of use/guidance of PIC with health promotion actions, both by professionals and by users, with an understanding of the PICs directed to the disease, treatment and cure^(^ 7 ^)^.

Thus, the present study aimed to understand the use of Integrative and Complementary Practices as a health promotion action.

## Method

**Figure 1:**
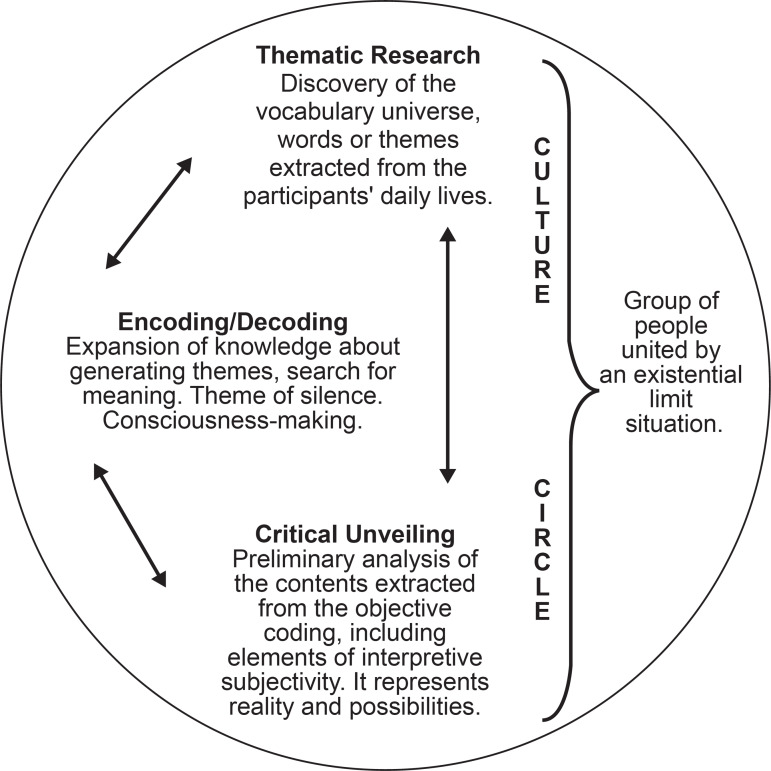
Scheme of Paulo Freire’s research itinerary

Source: Heidemann, et al., 2017^(^12^)^

A qualitative study, of the action-participant type^(^ 9 ^-^ 10 ^)^, was carried out using Paulo Freire’s Research Itinerary, which is based on a liberating pedagogical perspective, conducted through dialogue and horizontal relations. This methodological framework is organized into three dialectical moments: thematic investigation (data collection); encoding and decoding (data collection/data analysis); and critical unveiling (data analysis), which are outlined in Figure 1 below^(^ 11 ^-^ 12 ^)^.

The steps defined above are carried out in spaces called the Culture Circle. This is characterized by a group of people with some common interest, who discuss their problems and life situations, building a deeper perception of reality^(^ 11 ^-^ 12 ^)^.

The thematic research was carried out between the months from April to July 2017, in two Health Centers (HC) in the southern region of Brazil, these PHC services, organized according to the model of the Family Health Strategy (Estratégia Saúde da Família, ESF). Participants were professionals from the Family Health teams and the Family Health Support Center (Núcleo de Apoio à Saúde da Família, NASF). The Culture Yarning Circles were held on different days with each HC. The inclusion criteria were interest in participating, being a professional linked to the ESF or NASF and being present on the days of investigation. For exclusion, the following criteria were adopted: being a professional in another health service (secondary or tertiary care), be away for vacation or on leave during the thematic research period. To guarantee anonymity, the teams were identified by codename: Orient team and Occident team and the participants with PIC names.

The choice of locations occurred due to qualified intentionality of the HC, one that used the PIC in daily life and the other that was more focused on allopathic conceptions of assistance, from different health districts, in addition to establishing a relationship/bond before the beginning of the study.

Firstly, a contact was made with the CS, to present the research project and choose the participating team, which occurred based on the interest in the theme and availability. After the identification of the participating team, the days and times for the thematic investigation were agreed. Seven Culture Yarning Circles were developed, four with the Orient team and three with the Occident team, with an average duration of 60 minutes each yarning circle. Data were recorded in field diaries with notes from the main researcher. In order to improve the quality and fidelity of the investigated themes, audio recordings were made and transcribed in full, filming, and, photographic records, during the Culture Yarning Circles.

In both teams, in the first Culture Circle, the debate animator (researcher) recapitulated the objective and methodology of the study, explaining the stages of Paulo Freire’s Research Itinerary. Afterwards, the thematic investigation began, driven by a mandala that integrated three trigger questions: I) What do you think about PIC? II) How do you promote PIC in the HC and in the community? III) What are the easiness and difficulties to promote PIC in PHC? In order to provoke discussion among the participants, at first the members were divided into two small Yarning Circles, to then reconstruct the large Yarning Circle. The professionals were invited to represent the generating themes that emerged from the dialogues in written, drawn or magazine images. In the Orient team, 50 themes were highlighted and in the Occident team, 49.

In the second Culture Circle, the researcher took, in addition to the posters previously produced, one with the written organization of all the generating themes for the group to review, read, reflect on and start the stages of coding and decoding and critical unveiling. The Orient team codified three themes: I) Strengthening the SUS; II) Harm reduction; and, III) Comprehensiveness, according to Figure 2. The Occident team, in turn, also codified three generating themes: I) Work overload in SUS; II) Health promotion; and, III) Resistance times, which are described in Figure 3. At the end of the codification, the professionals expressed the order of priority for their discussion, considering the next two Culture Yarning Circles.

**Figure 2:** Representation of the codifications, with generating themes included in each coding and transversal theme. Orient Team

1. Strengthening the SUS* *(encoded, decoded and unveiled)*	2. Harm reduction *(encoded, decoded and unveiled)*	3. Seeing the person as a whole *(encoded, decoded and unveiled)*
1. Western Medicine 2. Oriental Medicine **3. Promote Health** **5. Music** 6. Health promotion, prevention and surveillance **7. Auriculotherapy** 10. Floripa: 100% of the population uses SUS* **11. Awareness** **12. Self-massage** **13. Garden** **14. Medicinal plants/teas** **15. Acupuncture** **16. Reiki** 17. Services Integration 18. Health Investment 20. Doctor-centered medicine 22. Time?/Clock? 23. Lack of health training 24. The complex health training 26. Community ignorance 27. Ignorance of professionals 28. Cost benefit 30. Autonomy 34. Everyone can be a healer 36. Information investment 39. Health is not a commodity 45. Decreased load on the health system 46. Comprehensiveness **47. Community garden** **48. Yoga** **49. Professionals and the community can apply integrative and complementary practices**	**3. Promote Health** **5. Music** **7. Auriculotherapy** 8. Health 9. Illness: 90% are not treated with drugs **11. Awareness** **12. Self-massage** **13. Garden** **14. Medicinal plants/teas** **15. Acupuncture** **16. Reiki** 19. Medicalization of Life 21. Pills 25. Mental health 29. Have no side effects 31. Increased longevity and chronic diseases 37. Naturals 38. They are part of SUS* **47. Community garden** **48. Yoga** **49. Professionals and the community can apply integrative and complementary practices**	**3. Promote Health** 4. Focus on the mind **5. Music** **7. Auriculotherapy** **11. Awareness** **12. Self-massage** **13. Garden** **14. Medicinal plants/teas** **15. Acupuncture** **16. Reiki** 25. Mental health 30. Autonomy 32. Indigenous population and their health practices 34. Everyone can be a healer 35. Complementary 40. Happiness 42. Integrative and complementary practices are for everyone 43. We will take good care of you 44. Treatment adherence 46. Comprehensiveness **47. Community garden** **48. Yoga** **49. Professionals and the community can apply integrative and complementary practices**

* SUS = Brazilian Public Health System

**Figure 3:** Representation of the codifications, with generating themes included in each coding and transversal theme. Occident Team

1. Work overload in SUS* *(encoded, decoded and unveiled)*	2. Health promotion *(encoded)*	3. Times of resistance *(encoded, decoded and unveiled)*
6. Teamwork 8. Qualified professional 9. Lack of time 10. Complaint/conduct 16. Some professionals make integrative and complementary practices 17. Some professionals do not make integrative and complementary practices 20. SUS*: kill a lion a day 21. In search of new paths **27. Health** 33. Overload 40. Professional: like an octopus 44. Quality in care 45. Work conditions 46. Expected response from SUS* 48. Lack of qualified training	1. Integration: 2. Popular knowledge for health promotion 4. New care demands 5. Plants/Nature 7. Innovation for whom? 22. Discover and understand 24. Happiness/joy **27. Health** 29. Innovation and access 31. Auriculotherapy 34. Community Health Agent: fundamental role 36. Service heart 37. Humanized portrait 38. Breastfeeding 42. Music/music therapy 43. Prevention of diseases 47. Circular dance 49. Circular dance wheel: experience that is working	12. Cultural resistance 13. Professional resistance 14. System resistance 15. Where is the doctors? 18. Interests at issue 25. Investment 26. Participation **27. Health** 28. Change of routine 30. Traditional knowledge 35. Medicalization/tablets 39. Professional/user relationship

* SUS = Brazilian Public Health System

The Orient team decided to talk at the third Culture Yarning Circle on Integrality and Harm Reduction within the scope of the PIC. The meeting was mediated by videos, reflections and discussions on the organization of work in PHC and the insertion of PIC in this scenario.

In the Occident team, the participants pointed out the themes Work overload in SUS and Times of resistance as central axes of the third Culture Yarning Circle. The researchers suggested videos on the topic and after viewing them, sheets and pens were given to the participants so that they could write their reflections, which were shared with the large group, in a continuous process of action-reflection-action about reality, decoding and unveiling the two proposed themes.

The method used^(^ 11 ^-^ 12 ^)^ allowed the adoption of different resources to conduct the third Culture Yarning Circle with the teams, based on the creative profile of each group, as it was noticed that the Orient team has the ability to more intense verbal expression, while the Occident team expresses itself more easily in written and drawn form.

In the fourth Culture Yarning Circle with the Orient team, the main researcher built a mandala with images and news about the SUS, given that the dialogues took place around the PICs as resources for the Strengthening of the SUS, highlighting powers and limits of the socio-political context- economic, inserting the PIC and an editorial against the collapse of SUS. In the Occident team, the fourth Yarning Circle did not happen, due to the demands of work that arose in the HC, so Health Promotion, a codified theme, was not decoded and unveiled.

It should also be noted that the dialogues/discussions in the Culture Yarning Circles had musical elements of oriental origin and mandalas, enabling a closer relationship with the area of PIC.

The generating themes (data) included in each codification and the transversal themes raised in the Culture Yarning Circles were organized in digital folders, classified by the Orient and Occident team. This organization would later enable the method of locating all the situations highlighted for the analysis of their contents (thematization) in the development of the research process.

The unveiling of the themes occurs concurrently with the thematic investigation, during the realization of the Culture Yarning Circles, according to the methodology of Paulo Freire^(^ 11 ^-^ 12 ^)^. For data analysis, the information obtained in the Culture Yarning Circles was carefully read and recorded in the respective folders. The elaboration of Figures 2 and 3 synthesized the data produced from each activity performed, articulating with the theme of the PIC. The highlighted data guided the reflection with the participants of the Culture Yarning Circle sand allowed the re-signification of the theme (new look) and the critical unveiling according to the Paulo Freire’smethodology^(^ 11 ^-^ 12 ^)^ performing the analysis in three significant themes^(^ 13 ^)^: Unveiling concepts and expanding the understanding of PIC; reducing damage to health and promoting comprehensiveness through PICs; and, PIC as a health promotion action in PHAPSC.

The research was developed according the ethical principles of resolution 466/2012, it was approved by the Research Ethics Committee of the Federal University of Santa Catarina with the opinion 1,828,562 and CAAE 61607316.4.0000.0121 of November 21, 2016.

## Results

The group of research participants was 30 health professionals, 18 from the Orient team and 12 from the Occident team. Regarding training, the following stand out: three doctors, three nurses, a dental surgeon, an oral health assistant, eight community health workers, a physical education professional and a psychologist. In addition, five residents (two doctors, a nurse, a physical education professional and a social worker) and seven academics (five in medicine and two in nursing).

In view of the codified generating themes, decoding and unveiling were promoted, in order to produce knowledge for the area under study.

In the first theme - “Unveiling concepts and expanding the understanding of PIC”, the professionals highlighted that PIC are part of the expanded concept of health. In some situations, they are complementary to allopathic practices, in others they are integrative, promoting comprehensive care and being the only therapy, and yet, they can be integrative and complementary as they were called by the National Policy: We follow Occident medicine, prescribing medicines. And the PICs lead to the vision of other practices, the happy person, without pain, walking, doing leisure, singing. For me, all PICs are integrative and complementary (Acupuncture); PICs have to do with the physical, the mental and the spirit, lead to human well-being (Anthroposophy); We think of PICs as a model of comprehensive care, which includes several types of knowledge besides the traditional one. We have to review this concept, as it seems that the PICs cannot support themselves, that they have to complement traditional knowledge and depending on the situation, PIC will be the only intervention (Circular Dance); It is another medical rationale, developed over the years. All PICs are integrative and complementary. There are communities that use ICPs as treatment, this is the main therapy (Reiki).

Therefore, depending on the situation, the PIC in use will be the person’s integral and unique treatment, the first choice of intervention, but in other cases, it will act to complement allopathy. It is noteworthy that regardless of whether it is an integrative practice, complementary or both, these therapies point to an emerging form of health care, which is solidified in essential values such as the rescue and perception of the human being, self-knowledge and the search for other ways of care.

Participants point out that PICs are an emerging form of attention and care in western society. They essentially work in the search for the understanding of human beings, of the ways of being and living, promoting health, quality of life, happiness and humanization of professionals and services: I perceive a search for new paths, a transition of models, which is recent and is in an adaptation period, both for professionals and the population (Ayurveda); PICs are a new demand for care. It is necessary to qualify the professionals. One care model does not discard the other, on the contrary, it integrates (Circular Dance).

With globalization and changes in the health-disease profile, new challenges impact the daily work in PHC. People seek health services due to different demands, which are often at a level of depth that requires more sensitive and humanized intervention strategies, with continuity of care in the medium and long term. PICs have an important potential to raise awareness of the transformation of professionals and users, promoting expanded and comprehensive care.

In this sense, the PIC as a paradigm of health care, allow other perspectives on the health-disease-care process, reaching all aspects that involve being, as the statements reveal: There is the question of the scientific, but also of how the person feels with a PIC. The circular dance group that we have at HC is an example, because it is difficult to maintain a group at SUS, and that group remains, and with more and more people wanting to participate (Circular Dance); PICs have a greater impact on pain, in acute situations, because most people come to HC with pain, anxiety problems, stress; today, this is the great demand (Yoga).

It is essential in the health sector and especially in PHC to understand the care strategies, in order to insert the PIC in individual and collective care, giving greater visibility to comprehensive, holistic and meaningful care within the social reality.

The second theme - “Reducing damage to health and promoting comprehensiveness through PICs”, revealed that PICs are resources that can be used to reduce damage to health amid the amazing scenario of the medicalization of life. In this context, it is possible to work with the awareness of individuals and families for the adoption of less invasive practices in facing daily adversities, promoting the rescue of familiar and popular knowledge and the decrease in the use of medicines: Harm reduction should be thought of for all people, because if I am avoiding a medication when doing a PIC, I am reducing health damage (Anthroposophy); PICs are a hodgepodge, a mixture of all cultures, spiritualities, which aim to reduce damage, reduce side effects, even as the population is aging and it is necessary to develop the autonomy of patients (Reiki).

Harm reduction can be promoted by PIC, as they encourage self-knowledge and the discovery of the best therapy for each individual. Thus, if it is not possible to adapt to a PIC, there are others that can be known and experienced, in search of effective care: Some people favor more than some practices and do not fit in with others. It all depends on what you are looking for, so it is important to have several alternatives, because people are different (Circular Dance); There is a type of PIC for each type of person. Harm reduction is in this sense, first seeing the patient’s need, then seeing which therapy is best (Yoga).

Integrality was unveiled as a result of the PIC which, due to their philosophy and way of understanding the human being, work in an integral dimension, uniting the physical body with the mental, emotional, spiritual, family and social. The approaches through the PIC encourage the realization of the positive concept of health, assigning an active role to users and involving them in the health-disease process in a conscious and responsible way: PICs naturally work with integrality, in the physical and psychological body, work with balance in everything (Homeopathy); PICs promote comprehensiveness, of seeing, treating, acting on the individual in all its aspects, psychic, social, spiritual, in his suffering, in his problem (Reiki); The PICs bring an integral view of the subject and his responsibility towards his health (Thermalism).

And in the third theme - “PIC as a health promotion action in PHC”, the dialogues produced in the Culture Circles, enabled reflections on the role of PICs, which, linked to the concepts of empowerment, autonomy and awakening to critical awareness, stimulate new horizons in health care. Health promotion seen as a strategy of happiness, well-being and quality of life, can be achieved through PIC, as evidenced by the following statements: How do we promote PIC in the HC and in the community? We do auriculotherapy in consultations, in groups. Auriculotherapy, music, dance, use of medicinal plants, community garden, acupuncture, reiki, self-massage, all improve the health of the population. To promote health is to see the person as a whole (Acupuncture); PICs focus attention on the person, promoting happiness. And we see the difference, difference in disposition, spirit, agility, flexibility, everything, in the physical, in the mental, in the way of living in society (Biodança).

Based on the understanding of health promotion, as one of the main pillars of PHC support, the differential of services that invest in this dimension is reinforced, with the purpose of effectively reaching human beings, generating health and working before the emergence of processes pathological, prioritizing the autonomy of individuals and families.

On the other hand, SUS professionals and users are faced with some difficulties to effectively promote PIC in their daily work, from different origins: Sometimes PIC is used, but the logic is not changed, the focus remains the disease (Circular Dance); One of the difficulties is precisely this ambiguous question, this confrontation between Western and Eastern medicine, so we need to educate ourselves for that (Reiki); People come to the HC and already know what they have, what they want, they just need a stamp and a signature. They don’t want to listen, they don’t want to understand, it’s a lot of medicalization (Shantala).

Therefore, it is necessary to invest in coping strategies, with sensitivity to awaken the real understanding of the role of PIC in PHC, involving professionals, users and managers in the search for knowledge, training and broadening of the view on health, as expressed by participant: From the moment you enter the PIC, you have to change the concept in relation to the person, change the thinking, because it is a different practice from the Occident (Yoga).

## Discussion

In the last decades, PICs have undergone an expansion process in Occident society, being inserted in the health systems of some countries, acting in the different dimensions of care, from health promotion and disease prevention to treatment, rehabilitation and cure^(^ 14 ^)^. PICs are understood as alternative, complementary and/or integrative practices to the therapies present in the current biomedical model^(^ 15 ^)^, which have a history and have the ability to be modified by social actors, presenting theoretical and practical continuity between past and present^(^ 16 ^)^, this makes them holistic in their action/intervention process.

On the other hand, few interventions use PICs to reduce health or illness problems or situations. A study carried out with pregnant women, in the United States, pointed out that pregnant women do not seek these complementary approaches because they do not know much about the subject and the professionals do not indicate them, even though these activities could provide benefits and care for maternal mental health during pregnancy^(^ 17 ^)^.

PICs as a health promotion action contribute to comprehensive care, especially with the worldwide increase in Chronic Noncommunicable Diseases. Although PICs emphasize health promotion and health care, research in this area is dominated by clinical aspects. The professionals who use them can use them as a health resource to increase the population’s access to certain preventive services integrated into the health system, but it becomes relevant to involve an interprofessional collaboration in order to seek to break the prejudices and overcome the differences in the perception of health and disease^(^ 18 ^)^.

The concept of care in an integral perspective considers light technologies, empowerment, co-responsibility, access, reception, resolution, fundamental factors to ensure the humanization of health practices. However, this is a long way to go, in view of the barriers imposed by the biomedical model, constituting a real daily challenge for teams, managers and users, working in the logic of comprehensive and universalassistance^(^ 19 ^)^. Comprehensiveness suggests the extension and development of care by different health professionals, in an expanded perspective, considering the human being in its multidimensionality, endowed with feelings, desires, afflictions and rationalities^(^ 20 ^)^.

There is evidence that the abusive use of medications harms individuals’ physical and mental health. There are a significant number of Brazilians who self-medicate, and, in addition to the inappropriate use of medications, many increase the dosages to accelerate the effect, putting health and quality of life at risk^(^ 21 ^)^. Poisoning and adverse drug reactions are currently a significant cause of hospitalization and mortality, standing out as a public health problem that puts people’s safety at risk. Analyzing the causes by gender, men died, mainly due to acute intoxication due to the use of multiple drugs and other psychoactive substances; and women, due to self-poisoning due to intentional exposure to anticonvulsants, sedatives, hypnotics, antiparkinsonian and psychotropic drugs. With regard to hospitalization, both genders had as their main cause acute poisoning by the use of multiple drugs and other psychoactive substances^(^ 22 ^)^.

In this perspective, the use of promotion, prevention and treatment strategies with ICP can lead to the comprehensive care of human beings and the reduction of damages resulting from the abuse of medicines. Scientific evidence found a reduction in the consumption of antibiotics and the incidence of recurrent infections, recovery time and sick leave, based on the use of PICs. However, conduct and protocols are needed to assist health professionals, as well as rigorous research to provide high-quality evidence before new guidelines can be developed, as there are differences in worldview between the biomedical model and the entire health system^(^ 17 ^)^.

Despite the expansion of PICs in PHC in recent decades, obstacles to sustaining this form of health care are faced between users and SUS professionals. Therefore, it is necessary to see knowledge and popular culture as a priority, enhancing discussions that foster the construction of strategies to strengthen PIC in PHC, for example, the investment in training for health professionals stands out^(^ 7 ^)^. The investment in the qualification of professionals, studies and promotion in the area is low, however, regardless of inducing resource, Brazilian municipalities offer PIC for health care, most of them with their own resources^(^ 23 ^)^.

In other countries it is no different, as in Spain, the education focused on PIC is insufficient, as there is no mandatory discipline in the curricula of nursing schools, consequently generating repercussions on the quality of care for future professionals^(^ 8 ^)^. For better health training, professionals and managers need more training, because they do not feel able to work within the scope of humanizing care practices, as there is a deficit of theoretical content during graduation and continuing education in work processes^(^ 19 ^)^. A care model that includes PIC promotes humanization, decreases costs with medicines and highly complex services. Therefore, it is necessary to promote the inclusion and development of academic spaces for training in this area^(^ 24 ^)^.

Worldwide, interest in the use of ICP has been gaining popularity, especially among children with cancer. In many countries, particularly in Africa, PICs have long been used within and outside the dominant health system, being used as the first and last resource for many diseases, in which cultural beliefs and practices lead to self-care, even when modern medicine is available. In high-income countries, the growing use of PICs has been linked to concerns about the adverse effects of chemical drugs and questions about traditional approaches^(^ 25 ^)^.

On the other hand, it is essential to overcome the health and care fragmentation model, highlighting the perspective of multidisciplinary and interdisciplinary work^(^ 19 ^)^. In this dimension, it is reaffirmed that health professionals who work with PICs encourage individuals to find their well-being and balance, as they understand that the body, as well as nature, has the capacity to seek stability for quality of life. PICs, as tools of care, when considering body, mind and spirit, promote health^(^ 26 ^)^, by instigating and recovering the notion of quality of life beyond illness, enhancing self-knowledge and (re)signifying knowledge in the face of the health-disease-care process. In this logic, the professional with a holistic view associated with these practices plays a fundamental role, demonstrating professional autonomy and competence, in all forms of performance^(^ 5 ^)^.

This research exposed important questions about health in PHC, allowing the understanding of the ESF and NASF teams, their conceptions and practices related to PIC as a health promotion action, bringing concerns, propositions, doubts and paths to the Culture Circles, confirming the potential for transformation through collective dialogue. Thus, the study contributed to the advancement of scientific knowledge in the field of nursing and health, as it caused changes and transformations through dialogues, from historical and conceptual issues of the PIC to practical issues of work organization, (re)thinking the form of care, for health promotion through these practices in the context of PHC.

The study’s limitations include: the time of contact with the participants, because the longer the time of contact, the greater the bond and the deepening of the discussions; and the difficulty of gathering professionals for the Yarning Circles, which is closely related to the current organization of health services. With regard to the continuity of investigations in this area of knowledge, the research group is working on other objectives, perspectives and with different target audiences, seeking to contribute with science and nursing, for the systematization of care through the PIC.

## Conclusion

PICs are resources that promote health, which rescue the essence of being, causing a more conscious thinking about life and the experiences of becoming ill, caring and curing, expanding the view of professionals towards integrality, considering human multidimensionality. In addition, PICs can be used to reduce damage resulting from the abuse of medicines and the consequent medicalization of life.
